# The Work of the New County Council

**Published:** 1913-03-15

**Authors:** 


### THE HEALTH OF LONDON.

### The Work of the New County Council.

Now that the strenuous election campaign is at an end, and the Municipal Reform Party have been returned to power on the London County Council with a considerably increased majority, it is worth while considering some of the work which lies before that body during the next three years in matters affecting the health of London. While there must inevitably be various changes in the constitution of the Council's committees the members of those committees which are more closely concerned with questions of public health have fared unusually well at the hands of the electors. Practically all the members of the Mental Hospitals Committee who sought re-election were successful, and this is particularly appropriate because the committee is one of the very few where " party " is entirely absent from its work. Mr. A. O. Goodrich, the chairman of the committee, whose strenuous work in the interests of the mental hospitals every member of the Council is willing to recognise, was again returned for Stepney. Mr. Whitaker Thompson, the vice-chairman, did not seek re-election, but most of the other members were again elected, although Mr. Frank Smith, the Labour representative on the committee, was defeated in North Lambeth. Both the chairman and the vice-chairman of the Public Health Committee, Major Cecil Levita and the Hon. Gilbert Johnstone, were returned. Mr. Johnstone was also the chairman of the Midwives Act Committee.

It will be interesting to see how the new Council deals with the ambulance question. The Council has already decided to apply this year for Parliamentary powers to co-ordinate the existing ambulance services, and when the question was last discussed at the Council it was stated that, if the proposal was found to be insufficient, no member of the Municipal Reform Party would grudge the expenditure necessary to equip London with a really efficient service. £15,000 a year will enable the County Council to do this efficiently, and it should be done forthwith. Most, if not all, of the party in power are pledged to take this step. Among those who in 1910 signed the postcard stating that they were in favour of the County Council establishing an efficient ambulance service in London similar to that now working in the City, the following were returned at last week's election: Mr. W. Warren, Battersea; Major Ernest Gray and Mr. W. Haydon, Brixton; Mr. R. A. Bray, North Camberwell; Mr. J. W. Domoney, City of London: Mr. R. M. Sebag-Montefiore, Clapham; Mr. H. E. A. Cotton and Mr. G. M. Gillett, East Finsbury; Miss N. Adler, Central Hackney; Mr. O. Warburg and Mr. G. W. H. Jones, North Hackney; Mr. T. Chapman, South Hackney; Mr. W. Reynolds, Hampstead; Mr. E. Smallwood, East Islington; Mr. George Dew, South Islington; Mr. F. Briant, North Lambeth; Mr. F. Carter, Lewisham; Mr. A. W. Yeo, Limehouse; Sir Edward White, West Marylebone; Mr. C. Stettauer, Mile End; Mr. F. St. John Morrow and Mr. C. U. Fisher, Norwood; Major Lewis-Barned, South Paddington; Mr. T. Gautrey and Lord Haddo, Peckham; Rev. J. Scott Lidgett, Rotherhithe; Mr. Harry Gosling and Mr. C. J. Matthew, St. George's-in-the-East; Mr. A. W. Claremont, East St. Pancras; Mr. J. C. Denison Pender, South St. Pancras; Mr. J. A. Dawes, M.P., and Mr. C. Jesson, Walworth; and Mr. R. W. Granville-Smith, Westminster.

The Mental Hospitals Committee will find itself faced with plenty of work in view of the fact that the building of the Council's eleventh mental hospital is to be pushed forward without delay, and the erection of the Maudsley Mental Hospital will also engage their attention immediately. The need for the new mental hospital is emphasised by a report just issued by the committee which shows that the number of lunatics under certificate in London County mental hospitals or in other county or borough mental hospitals or licensed houses under contract made by the Boards of Guardians and of lunatics in workhouses or with friends at the beginning of the year was 28,638, which, compared with the total of 28,011 last year, gives an increase of 627, the largest recorded since 1910. Of the total, the County is responsible for finding accommodation for 20,931, an increase of 502.

The work of the Public Health Committee has been largely increased owing to the provision of sanatorium benefit by the Insurance Act, and one of the most important tasks which faces the committee in the near future is the preparation of a complete scheme for the treatment of tuberculosis in London, the committee having come to the conclusion that the work of the various authorities and institutions in this matter must be co-ordinated. With this object in view conferences are to be held with the representatives of the various interests concerned, and the committee hope to be in a position shortly to present to the Council a scheme which will enable all interested in the provision of treatment for tuberculosis in London to decide what arrangements to make for the future treatment of the disease.

The return of the Municipal Reform Party to power means that there is little prospect of establishing a system of school clinics during the next few years, as that party consider that the medical treatment of school children is better obtained at existing hospitals of the London Hospital type. The public are likely to hear a great deal of this subject, for the Progressives have always fought, and, even with their diminished numbers, will continue to fight, for the establishment of a complete system of school clinics. It will be seen that there is plenty of public health work to occupy the attention of the Council during their term of office, and it is to be hoped that now that the elections are at an end the Council will remember that first and foremost it is an administrative body, and that there is no need for party considerations to enter into these questions.

